# Obesity/Overweight as a Meaningful Modifier of Associations Between Gene Polymorphisms Affecting the Sex Hormone-Binding Globulin Content and Uterine Myoma

**DOI:** 10.3390/life15091459

**Published:** 2025-09-17

**Authors:** Marina Ponomarenko, Evgeny Reshetnikov, Maria Churnosova, Inna Aristova, Maria Abramova, Vitaly Novakov, Vladimir Churnosov, Alexey Polonikov, Mikhail Churnosov, Irina Ponomarenko

**Affiliations:** 1 Department of Medical Biological Disciplines, Belgorod State National Research University, 308015 Belgorod, Russia; 1256888@bsuedu.ru (M.P.); reshetnikov@bsuedu.ru (E.R.); churnosovamary@gmail.com (M.C.); aristova@bsuedu.ru (I.A.); abramova_myu@bsuedu.ru (M.A.); 659864@bsuedu.ru (V.N.); 958561@bsuedu.ru (V.C.); polonikov@rambler.ru (A.P.); ponomarenko_i@bsuedu.ru (I.P.); 2 Department of Biology, Medical Genetics and Ecology, Research Institute for Genetic and Molecular Epidemiology, Kursk State Medical University, 305041 Kursk, Russia

**Keywords:** sex hormone-binding globulin, uterine myoma, obesity/overweight, single nucleotide polymorphism

## Abstract

The main goal of this study was to consider the role of obesity/overweight as a potential modifier of associations between gene single nucleotide polymorphisms (SNPs) affecting the sex hormone-binding globulin level (SHBG_level_) and uterine myoma (UM). In the two women cohorts differentiated by body mass index (BMI) (BMI ≥ 25, n = 782 [379 UM/403 control] and BMI < 25, n = 760 [190 UM/570 control]), the association of genome-wide association studies (GWAS)-correlated SHBG_level_-tied nine loci with UM was studied by method logistic regression with a subsequent in-depth evaluation of the functionality of UM-causal loci and their strongly linked variants. BMI-conditioned differences in the associations of SHBG_level_-tied loci with UM were revealed: in the BMI < 25 group, a variant rs17496332 (A/G) *PRMT6* was UM-correlated (OR = 0.70; *p_perm_* = 0.024), and in the BMI ≥ 25 cohort, a SNP rs3779195 (T/A) *BAIAP2L1* was UM-associated (OR = 1.53; *p_perm_ =* 0.019). Both the UM-causal loci and their proxy SNPs have pronounced probable functionality in the organism as a whole, as well as in the liver (the SHBG synthesis place), adipose tissue, uterus, etc., thereby influencing significant processes for UM biology such as regulation of the gene transcription, embryogenesis/development, cell proliferation/differentiation/apoptosis, metabolism, lipid exchange, etc. In conclusion, the results of our work demonstrated, for the first time, the essential role of obesity/overweight as a meaningful modifier of associations between SHBG_level_-tied polymorphisms and UM.

## 1. Introduction

Uterine myoma (UM) is the most common benign tumor among women of reproductive age [1,2]. Clinical manifestations of UM, such as heavy menstrual bleeding causing anemia/chronic fatigue, pelvic discomfort, decreased fertility, and pregnancy complications, significantly reduce women’s life quality [3,4,5]. In many countries of the world, UM is the leading indication for hysterectomy [6,7,8]. The healthcare system’s cost for the treatment of UM patients is very high and amounts to USD 34.4 billion annually in the United States, USD 348 million in Germany, USD 120 million in France and USD 86 million in England [9]. Thus, UM is a global problem for both the health system and a meaningful proportion of women due to a significant decrease in their life quality, which takes this disease beyond a purely gynecological problem.

The genetic basis of UM is being actively studied in various countries around the world [10,11,12,13,14,15]. A substantial role of heredity has been shown (up to 69%) in the occurrence of UM [16]. The risk of developing UM among first-degree relatives of UM-affected women exceeds the average population value by 2.5 times [17]. The number of polymorphic variants associated with UM ranges from several dozen (GWAS data) to several hundred (data from associative genetic studies) [3,18,19,20,21,22,23,24,25,26,27,28]. Along with this, despite the considerable material accumulated on the issue of the UM genetic foundations, a relatively small proportion of the heredity of the disease (less than 1/5, 13% of 69%) can be explained by the available data from associative studies (SNP heredity, GWAS data) [21], which is extremely insufficient and requires continued further research into the genetic basis of UM.

One of the significant risk factors for UM is BMI [29,30,31,32,33,34]. Based on the analysis of GWAS data by the Mendelian randomization (MR) method, an increased UM risk was shown with an increase in both BMI (OR = 1.13) [32] and BMI-related indicators such as waist circumference (OR = 1.16–1.93) and hip circumference (OR = 1.06–1.10) [30]. A direct correlation between BMI and UM has also been confirmed in the largest meta-analyses (OR = 1.19) [29]. It is believed that in “excess” adipose tissue there is an increased conversion of androgens into estrogens, which stimulates the UM development, and the SHBG production decreases, which leads to an increase in the level of UM-stimulating free androgens and estrogens [29]. A marked decrease in SHBG_level_ (by 6–35%) and significant increase in the content of estrone (21–34%), estradiol (by 45–68%), free (bioactive) fractions of testosterone (35%), and estradiol (101%) with an increase in BMI in women have been convincingly shown in experimental studies [35].

So, at this point in time, it is obvious that, firstly, BMI is an important UM risk factor [29,30,32]. Secondly, BMI has a significant effect on the SHBG level and SHBG-related sex hormones (testosterone and estrogens) [35,36], which is important for UM pathophysiology [29,37]. Thirdly, SHBG_level_ is genetically determined and there are numerous GWAS data confirming this [38,39,40,41,42,43,44]. Based on the above, it is highly likely that BMI may be a meaningful modifier of associations with gene polymorphisms affecting the SHBG_level_ with UM. Importantly, at this point in time, there are no data on the effect of BMI/obesity/overweight on the nature of SHBG_level_-related polymorphisms associated with UM, and our work is the first in the world devoted to this issue.

## 2. Materials and Methods

### 2.1. Study Subjects

The present study was performed in two women cohorts, differentiated by BMI (BMI ≥ 25, n = 782 [379 UM/403 control] and BMI < 25, n = 760 [190 UM/570 control]). The issues of the organization/conduct of the research were considered/supported at the meeting of the specialized (medical) Ethics Committee of Belgorod State University. When forming the sample, each subject (UM/UM-free) confirmed her consent to participate in this study with a personal signature. To be included in the study, the woman had to be of Russian nationality and born in the Central region of the Russian Federation [45,46]. Diagnosis of UM in patients was performed by certified gynecologists in the specialized (gynecological) department of the Belgorod Perinatal Center based on the morphologist’s conclusion after examining UM samples obtained as a result of hysterectomy. To be included in the control group, the mandatory conditions were that the woman did not have any (anamnestic/clinical/ultrasound) signs indicating pathology of the pelvic organs (UM/adenomyosis/endometriosis/endometrial hyperplasia). The necessary examination of the control group women was performed at the Belgorod Perinatal Center during periodic (annual) medical examinations. The presence of pelvic and breast cancers, as well as severe diseases of vital organs in a woman, was an absolute indication for exclusion from the study. The main phenotypic characteristics of the studied UM/UM-free groups in BMI ≥ 25/BMI < 25 cohorts are shown in Table 1. In both the BMI ≥ 25/BMI < 25 cohorts, differences in “UM vs. UM-free” were found in several parameters such as age, number of pregnancies/births, infertility history, family history, and induced abortions; also, in the BMI ≥ 25 group, additional “UM vs. UM-free” differences in BMI and a history of chronic endometritis were found. Based on these results, the above parameters were included in the genetic calculations when studying “UM-SHBG_level_-tied SNPs” associations as covariates.

### 2.2. SNP Selection/Detection

We genotyped nine SHBG_level_-tied loci according to previously performed GWAS (Supplementary Table S1 [38,39,42,43,44]) with functional relevance (Supplementary Table S2; HaploReg data-v.4.2, accessed: 10 November 2024 [47]), such as rs12150660 (G>T) *SHBG*, rs17496332 (A>G) *PRMT6*, rs7910927 (G>T) *JMJD1C*, rs780093 (C>T) *GCKR*, rs8023580 (T>C) *NR2F2*, rs3779195 (T>A) *BAIAP2L1*, rs10454142 (T>C) *PPP1R21*, rs4149056 (T>C) *SLCO1B1*, and rs440837 (A>G) *ZBTB10*.

DNA samples taken for genotyping were (a) previously isolated from venous blood (the phenolic chloroform technique was used) and (b) stored in kelvinators at a temperature of −80 °C. (c) The necessary purity parameters were available [the compliance index “260/280 Nm” corresponded to the 1.7–2.0 values] [48] and changes were performed on a Nano-Drop-2000 (Thermo Fisher Scientific, Waltham, MA, USA). Genotyping of polymorphic loci was performed using polymerase chain reaction (PCR) by allele discrimination using sets specially synthesized for the present study. The genotyping kits were developed/produced by the R&D company TestGene, specialized in the field of genetic research (https://testgene.com/). Data on the sequences of oligonucleotide primers/probes used for genotyping SNPs of candidate genes are presented in Table 2. A CFX96 device was used for PCR [49]; the amplification conditions were set according to the instructions provided by the developer. Genotype identification was performed using certified CFX-Manager™ software (version 3.1). When carrying out SNP genotyping, quality control of the obtained genetic data was accomplished using the repeated genotyping procedure of a sample of a certain part of DNA (5–6%) (so-called blind genotyping) [50]. As a result of this procedure, a match was achieved in more than 99% of cases in the “first/re-genotyped” results, which suggests that the experimental (genetic) data obtained were of sufficient quality.

### 2.3. Statistical/Bioinformatics Genetic Data Analysis

Associations of SHBG_level_-tied loci with UM were studied in both the examined women cohorts (BMI < 25/BMI ≥ 25). In this regard, the indicator for the identification of statistically significant associations was adjusted by us to the level of “*p_bonferroni_* ≤ 0.025” (Bonferroni correction was used, which took into account the number of groups being compared and was equal in our case to 2 [51]). The UM-SNP relationship was evaluated in the gPLINK program [52] using logistic regression (such genetic models were considered as additive, dominant, recessive, allelic [53]). Association indicators (OR; 95%CI) were adjusted for the necessary covariates (age, BMI, number of pregnancies/births, infertility history, family history, induced abortions, chronic endometritis history) and permutation procedures were performed (in order to correct false positive results when evaluating associations of multiple SNPs with UM [54,55,56]). For statistically significant association indicators (corresponding to parameter “*p_perm_* ≤ 0.025”), the “power” value was calculated (the Quanto program was used [57]).

For two polymorphisms, rs17496332 (A/G) *PRMT6* and rs3779195 (T/A) *BAIAP2L1*, which showed significant associations with UM, an in depth in silico analysis of functionality [58,59,60] was performed (not only two UM-causal loci, but also proxy SNPs [r^2^ ≥ 0.8] [61,62,63,64] were considered) using such bioinformatic resources/databases as HaploReg (v.4.2, accessed: 10 November 2024 [47]; GTExportal (accessed: 12 June 2024) [65]; STRING (accessed: 13 December 2024) [66].

## 3. Results

In the two women cohorts differentiated by BMI [BMI < 25/BMI ≥ 25] for 9 studied polymorphisms, when Bonferroni correction was introduced (the generally accepted level of statistical significance equal to *p* < 0.05 was adjusted for the number of studied loci [n = 9], *p_bonferroni_* < 0.05/9 < 0.006), the HWE rule holds true (BMI < 25:0.176 ≤ *p_HWE_* ≤ 1.000 [UM] and 0.066 ≤ *p_HWE_* ≤ 0.926 [control] (Supplementary Table S3); BMI ≥ 25:0.016 ≤ *p_HWE_* ≤ 1.000 [UM] and 0.030 ≤ *p_HWE_* ≤ 0.900 [control] (Supplementary Table S4)).

BMI-conditioned differences in the association of SHBG_level_-tied loci with UM were revealed: in the BMI < 25 group, a variant rs17496332 (A/G) *PRMT6* was UM-correlated (AA vs. AG vs. GG [additive model]; OR = 0.70; 95%CI = 0.51–0.94; *p* = 0.023; *p_perm_* = 0.024; power = 80.96%), and in the BMI ≥ 25 cohort, a SNP rs3779195 (T/A) *BAIAP2L1* was UM-associated (AA + TA vs. TT [dominant model]; OR = 1.53; 95%CI = 1.06–2.09; *p* = 0.018; *p_perm_* = 0.019; power = 80.91%) (Table 3). So, the above-stated data testify that the allele A rs3779195 (T/A) *BAIAP2L1* increases the risk of UM (by more than 50%), and the allele G rs17496332 (A/G) *PRMT6*, on the contrary, reduces the UM risk (by 15% for each allele G).

### 3.1. Probable Functionality of the UM-Associated Loci (In Silico Data)

**Figure 1:**
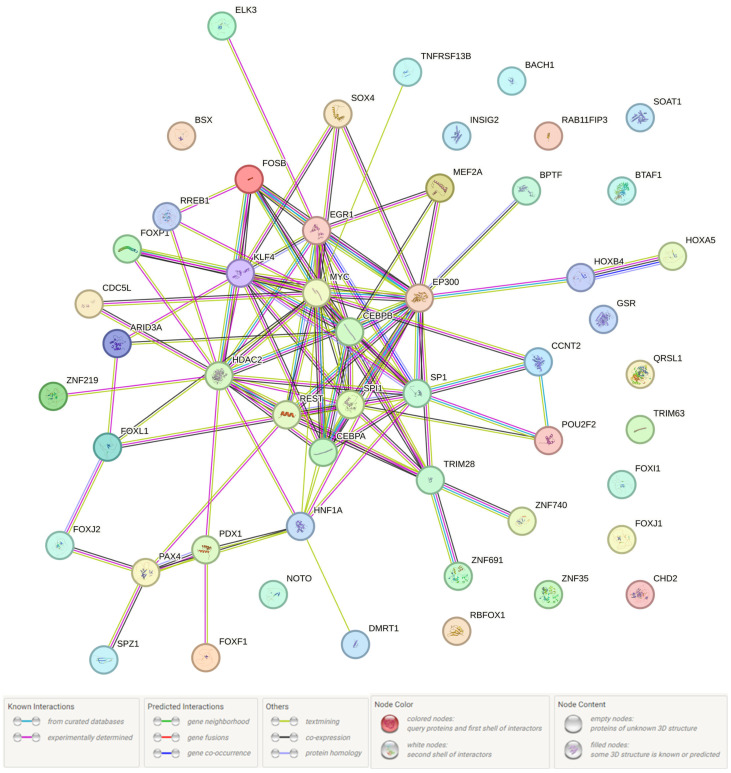
Network of transcription factor interactions and PRMT6 protein associated with UM risk, mediated by rs17496332 (A/G) *PRMT6* and proxy SNPs (STRING data). Detailed information on the molecular/biochemical functions of the TFs/protein functionally related to the UM-associated rs17496332 (A/G) *PRMT6* and their proxy 14 SNPs is provided in Supplementary Table S12 (according to the STRING data).

Having identified BMI-conditioned differences in the involvement of SHBG_level_-tied loci in UM susceptibility, in this work section we attempted to find, using the in silico methodology, biological mechanisms (modifications of epigenetic status, gene expression and splicing, protein interactions, pathways) that can determine these features. To do this, we examined the probable functionality in the organism (as a whole), the liver (the SHBG synthesis place [67,68]), adipose tissue (according to our above-stated results, BMI is a considerable modifier of genetic associations), and uterus (the target organ for UM) of UM-associated loci in groups with different BMI, such as rs17496332 (A/G) *PRMT6* [BMI < 25] and rs3779195 (T/A) *BAIAP2L1* [BMI ≥ 25] and their proxy loci (14 SNPs and 20 SNPs appropriately). The data obtained as a result of this analysis are shown in Table 4 and Table 5, Figure 1 and Supplementary Tables S5–S9.

### 3.2. The Presumed UM-Associated Functionality in the BMI < 25 Women Group rs17496332 (A/G) PRMT6

SNP rs17496332 (A/G) *PRMT6* and all 14 proxy loci have potential functionality (Table 4 and Supplementary Tables S5–S7). UM-causal variant rs17496332 (A/G) *PRMT6* affects the genome interaction in the region of the *PRMT6* gene (position 53 kb 5′) with two transcription factors (TFs) such as DMRT1 and FAC1 (Supplementary Table S5). Herewith, the UM-protective allele G of this SNP significantly reduces the DNA affinity to TF DMRT1 (the difference in LOD_score_ parameters between G (2.1) and A (12.8) alleles was ΔLOD_score_ = −10.7) and increases its affinity to TF FAC1 (ΔLOD_score_ = +1.7). Also, 13 out of 14 LD loci exert the interaction of the *PRMT6* gene regulatory region with 57 TFs such as AP-1, Arid3a, Bach1, Brachyury, Bsx, CACD, Cart1, CCNT2, Cdc5, CEBPA, CEBPB, CHD2, Egr-1, Ets, GR, Foxf1, Fox, Foxa, EWSR1-FLI1, Foxi1, Foxj1, Foxj2, Foxl1, Foxp1, GATA, HDAC2, HNF1, Hoxa5, Hoxb4, Ik-2, IRC900814, Irf, KAP1, Klf4, Mef2, Myc, NF-AT, Sox, NRSF, p300, Pax-4, Pdx1, Pou2f2, PU.1, RREB-1, RORalpha1, SP1, Spz1, SREBP, STAT, TATA, Zfp105, UF1H3BETA, Zfp281, Zfp691, Zfp740, and ZNF219 (Table 4). Two proxy loci, such as rs111232683 and rs4914939, have been involved in the regulation of DNA contact with the largest number of TFs (21 and 15 TFs appropriately) (Supplementary Table S5). So then, in total, the UM-causal variant rs17496332 (A/G) *PRMT6* and its 13 proxy loci determine the cooperation of the near *PRMT6* gene genome region with 59 TFs (Table 4).

SNP rs17496332 (A/G) *PRMT6* and 9 LD variant affect the *PRMT6* expression in different organs (>20), including organs implicated in both the SHBG formation (liver) and in the UM biology (thyroid, adrenal, brain, blood, etc.) (Supplementary Tables S6 and S7). The influence of these polymorphisms (UM-causal locus and its 9 LD SNPs) on *PRMT6* gene expression in adipose tissue (both visceral and subcutaneous) is extremely important (this feature can also determine BMI-conditioned differences in the natural association of this locus with UM). In all of the above organs, the UM-protective allele G was linked with low *PRMT6* transcriptional activity. Interestingly, three proxy loci such as rs3861909, rs72697623, and rs4914939 were localized in the potential enhancers area of the fat cells (the adipose-derived mesenchymal stem cell cultured cells) (Table 4).

Next, we studied the interaction of 59 TFs and PRMT6 protein functionally related to the rs17496332 (A/G) *PRMT6* and their proxy 14 SNPs. After conducting this analysis in the STRING program (Figure 1), we have identified the most major communications (0.958 ≤ score ≤ 0.999) between such TFs as SP1-EP300, MYC-EP300, EP300-CEBPB, SPI1-CEBPA, CEBPB-CEBPA, MEF2A-EP300, FOSB-EGR1, SP1-MYC, REST-HDAC2, and MYC-CEBPB. Among the many biological pathways in which the UM-impact TFs/protein interactions have been involved, the following processes prevail: (1) gene transcription regulation; (2) embryogenesis/development; (3) cell proliferation/differentiation/apoptosis (including smooth muscle cells) regulation; and (4) metabolism (including lipid exchange) regulation (Supplementary Table S10).

### 3.3. The Supposed UM-Associated Functionality in the BMI ≥ 25 Women Group rs3779195 (T/A) BAIAP2L1

Locus rs3779195 (T/A) *BAIAP2L1* and all 20 LD variants have the expected functionality (Table 5, Supplementary Tables S5–S9). UM-causal SNP rs3779195 (T/A) *BAIAP2L1* coordinates the DNA “collaboration” in the *BAIAP2L1* and *BRI3* genes region with TF Foxp1, and herein, the UM-risk allele A of the above SNP increases the affinity of this genome site with Foxp1 (ΔLOD_score_ = +0.9) (Supplementary Table S5). Importantly, 17 out of 20 LD variants exert the interaction of the regulatory region of *BAIAP2L1/BRI3* genes with 85 TFs and regulatory proteins such as AP-1, AP-2, AP2ALPHA, AP2GAMMA, Arid3a, Ascl2, Bach1, Bach2, BAF155, BATF, BHLHE40, CEBPB, CHD2, CEBPG, CMYC, CTCF, CTCFL, Dbx1, DMRT4, E2F, EBF, Egr-1, FAC1, Foxl1, GTF2F1, RAD21, GABP, GATA, GR, HDAC2, HMGN3, HNF1, HNF4, Hoxa10, Hoxa4, Hoxa9, Hoxb13, KAP1, Hoxd10, Lhx3, Lmo2-complex, MAX, MAFK, MAZ, MAZR, Mef2, MXI1, Myc, MZF1:1–4, Ncx, POL24H8, NF-kappaB, Nkx2, Nkx3, Nr2f2, Nrf1, NRSF, p300, Pax-2, Pax-4, Pax-6, PLZF, POL2, Pou2f2, SMC3, PU1, Pou3f2, Sin3Ak-20, PRDM1, Pou6f1, RXRA, Sox, SRF, STAT, TATA, VDR, TCF12, TCF4, UF1H3BETA, USF1, Zfp105, Zfp161, Znf143, SP1, and ZNF263 (Table 5). Interestingly, five proxy variants, such as rs13232861, rs3779196, rs11290747, rs6967728, and rs6950023, coordinate DNA communication with the maximum number of TFs/regulatory proteins (22, 14, 12, 12, 11 appropriately) (Supplementary Table S5). So, in summary, the UM-causal SNP rs3779195 (T/A) *BAIAP2L1* and its 17 LD variants define the relationship of the genome position at near *BAIAP2L1* and *BRI3* genes with 86 TFs (Table 5).

It is extremely important to have the expected functionality of several proxy loci in the liver, the main place of SHBG synthesis in the organism, including their localization in the regulatory elements of the genome such as potential enhancers (9 SNPs:rs6950023, rs6967728, rs77032872, rs7015, rs1688607, rs11290747, rs3779196, rs6965424, rs10953259) and promoters (2 SNPs:rs6950023, rs6967728), active enhancers (7 SNPs:rs6950023, rs6967728, rs77032872, rs13232861, rs11290747, rs12704986, rs3779196) and active promoters (5 SNPs:rs6950023, rs6967728, rs11290747, rs3779196, rs10953259) (Table 5). Also, the UM-causal locus rs3779195 (T/A) *BAIAP2L1* and its 17 highly linked variants have been correlated with *RP11-307C18.1* and *BRI3* gene expression in the liver (Table 5): UM-risk allele A rs3779195 was associated with a reduced eQTL of *RP11-307C18.1* [NES = −0.54] and an enlarged eQTL of *BRI3* [NES = 0.87] in the liver (Supplementary Table S6). Interestingly, the UM-causal locus rs3779195 (T/A) *BAIAP2L1* and its 17 LD SNPs (Table 5) have a meaningful eQTL effect (in relation to the *RP11-307C18.1* gene) in the target organ of the disease we are considering—the uterus (UM-risk allele A was correlated with lowered *RP11-307C18.1* transcription [NES = −0.84]) (Supplementary Table S6). Also, SNP rs3779195 (T/A) *BAIAP2L1* and 17 proxy variants affect gene expression (15 genes: *AC004967.7*, *ASNS*, *BAIAP2L1*, *BRI3*, *LMTK2*, *TECPR1*, *RP11-307C18.1*, *RP11-307C18.2*, *RP11-307C18.3*, *RP11-307C18.4*, *RP11-307C18.5*, *RP11-307C18.6*, *RP11-307C18.7*, *RP11-307C18.10*, *RP11-307C18.11*) (Supplementary Tables S6 and S7) and splicing (3 genes: *BRI3*, *TECPR1*, *BAIAP2L1*) (Supplementary Tables S8 and S9) in different organs such as the ovary (*RP11-307C18.1* [eQTL]), thyroid (*RP11-307C18.1*, *BAIAP2L1*, *TECPR1*, *LMTK2*, *BHLHA15* [eQTL] and *BRI3* [sQTL]), adrenal gland (*RP11-307C18.1* [eQTL]), brain (*RP11-307C18.1*, *BHLHA15* [eQTL] and *BRI3*, *TECPR1* [sQTL]), blood (*RP11-307C18.1*, *TECPR1* [eQTL]), skeletal muscle (*RP11-307C18.1*, *BRI3*, *BAIAP2L1*, *ASNS* [eQTL] and *BRI3* [sQTL]), etc. (>20), implicated in the UM biology.

We have identified the adipose-impact functionality of the UM-causal locus rs3779195 (T/A) *BAIAP2L1* and a number of its proxy variants. Thus, the UM-associated SNP rs3779195 (T/A) *BAIAP2L1* affects the two genes’ expression (*RP11-307C18.1* and *BRI3*) and the *BRI3* gene splicing in both visceral and subcutaneous adipose tissue (Table 5). Meanwhile, the UM-risk allele A rs3779195 correlates with low expression of both above genes (*RP11-307C18.1* and *BRI3*) and low levels of *BRI3* gene splicing in both visceral and subcutaneous adipose (Supplementary Tables S6 and S7). Adipose-impact eQTL (*RP11-307C18.1* and *BRI3*) and sQTL (*BRI3*) effects were additionally registered by us for 17 strongly linked loci (Table 5). Also, a number of proxy loci (5 out of 20 SNPs) exhibit significant epigenetic effects (located in the area of potential enhancers/promoters, active enhancers/promoters) in various fat cell cultures such as mesenchymal stem cell-derived adipocyte cultured cells (rs6950023, rs6967728, rs77032872), adipose-derived mesenchymal stem cell cultured cells (rs6950023, rs6967728, rs77032872, rs7015), and adipose nuclei (rs6950023, rs6967728, rs77032872, rs7015, rs1688607) (Table 5).

**Figure 2:**
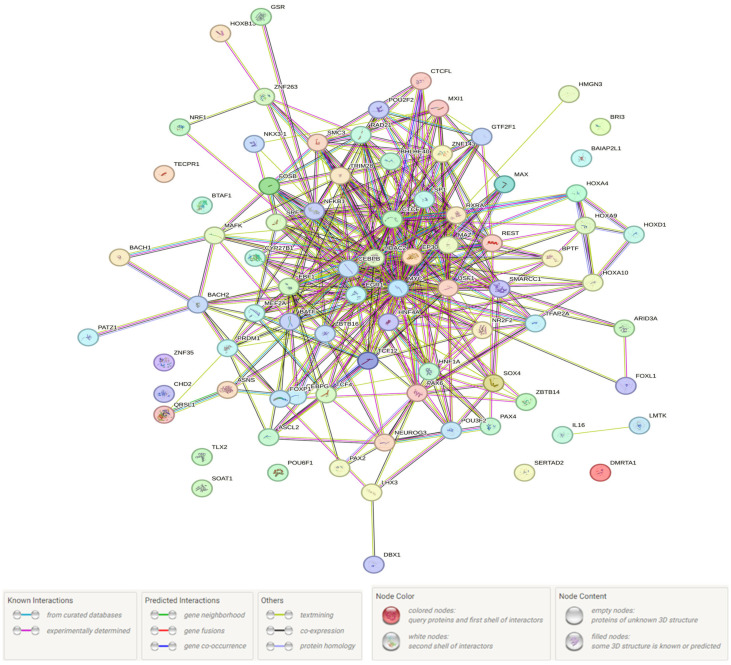
Network of interaction at 86 protein-regulatory/TFs and 15 protein products associated with UM risk, mediated by rs3779195 (T/A) *BAIAP2L1* and proxy SNPs (STRING data). Detailed information on the molecular/biochemical functions of the TFs/proteins functionally related to the UM-associated rs3779195 (T/A) *BAIAP2L1* and their proxy 20 SNPs is provided in Supplementary Table S13 (according to the STRING data).

In conclusion, we examined the “joint work” of 86 protein-regulatory/TFs and 15 protein products of genes functionally related to rs3779195 (T/A) *BAIAP2L1* and their proxy 20 SNPs (ultimately, the collaboration of 101 different proteins was studied). According to the results, presented in Figure 2, impact links (0.989 ≤ score ≤ 0.999) were recorded between such TFs/protein-regulators as SMC3-RAD21, SP1-EP300, RAD21-CTCF, MYC-EP300, MYC-MAX, NFKB1-EP300, MXI1-MAX, EP300-NFKB1, EP300-CEBPB, TCF4-TCF12, SMC3-CTCF, and MEF2A-EP300, MAFK-BACH1. Lot pathways have been identified in which UM-related TFs/protein-regulators/proteins interactions were involved, among which the following main groups can be distinguished: (1) gene transcription regulation; (2) glucose homeostasis regulation; (3) sex hormone pathways; (4) embryogenesis/development; (5) cell proliferation/differentiation/apoptosis regulation; (6) metabolism (including lipid exchange) regulation; and (7) vitamin D metabolism (Supplementary Table S11).

## 4. Discussion

**Figure 3:**
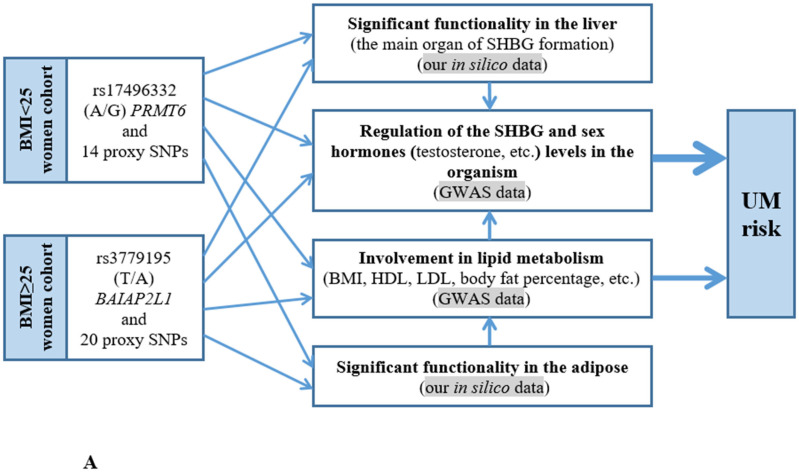

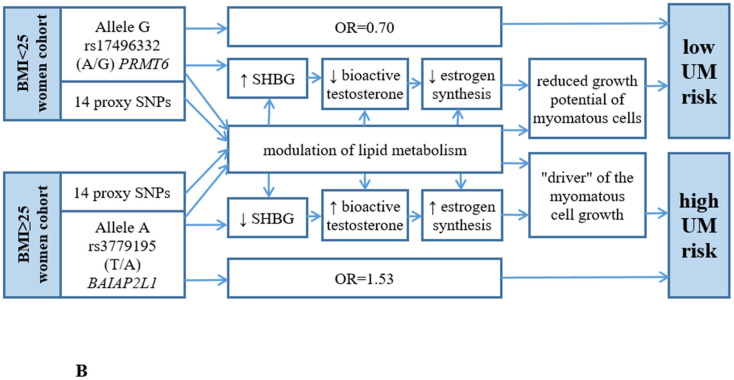
Potential biological mechanisms (**A**) and orientation (**B**) of involvement in the SHBG_level_-tied SNPs (rs17496332 (A/G) *PRMT6* [BMI < 25] and rs3779195 (T/A) *BAIAP2L1* [BMI ≥ 25]), in UM risk in women with different BMIs. The arrows indicates increased or decreased hormone levels.

The results of our work demonstrated, for the first time, the essential role of obesity/overweight as a meaningful modifier of associations between SHBG_level_-tied polymorphisms and UM: rs17496332 (A/G) *PRMT6* was UM-correlated in BMI < 25 group and rs3779195 (T/A) *BAIAP2L1* was UM-associated in BMI ≥ 25 cohort. Both UM-causal loci and their proxy SNPs have pronounced probable functionality in the organism as a whole, the liver (the SHBG synthesis place), adipose tissue (according to our above-stated results, BMI is a considerable modifier of genetic associations), uterus, etc., thereby influencing such significant processes for UM biology as the regulation of gene transcription, embryogenesis/development, cell proliferation/differentiation/apoptosis, metabolism, lipid exchange, etc. Importantly, in the cohort we studied, obesity/overweight was a significant risk factor for UM (OR = 2.82, 95%CI 2.26–5.52, *p* = 0.0005 [63]) and can be a meaningful modifier of associations between gene polymorphisms affecting the SHBG_level_ and UM, which we identified for the first time in the world in our study. Potential biological mechanisms and orientation of involvement in the SHBG_level_-tied SNPs (rs17496332 (A/G) *PRMT6* [BMI < 25] and rs3779195 (T/A) *BAIAP2L1* [BMI ≥ 25]), in UM risk in women with different BMIs, are presented in Figure 3.

As our results showed, the allele G rs17496332 (A/G) *PRMT6* reduces the UM risk (by 15% for each allele G) in women with BMI < 25 (OR = 0.70). The UM-causal locus rs17496332 (A/G) *PRMT6* and its proxy SNPs determine the cooperation of the near *PRMT6* gene genome region with 59 TFs, affect the *PRMT6* expression in different organs (>20), including organs implicated in both the SHBG formation (liver) and in the UM biology (thyroid, adrenal gland, brain, blood, etc.), and have adipose-impact functionality (several SNPs were localized in the potential enhancers area of the fat cells, affecting *PRMT6* gene expression in both visceral and subcutaneous adipose). The GWAS materials, presented by Coviello et al., inform us about the connection between the rs17496332 (A/G) *PRMT6* and SHBG_level_: the major allele A marks a reduced SHBG_level_ (β = −0.028, *p* = 1 × 10^−11^) and, accordingly, the minor allele G marks an increased SHBG_level_ [39]. So, the SHBG-boosting allele G rs17496332 has been associated with a higher SHBG_level_ (Coviello et al. GWAS result [39]) and a low risk of UM (our data [OR = 0.70]). Interestingly, some loci, strongly linked with UM-causal SNP rs17496332 (A/G) *PRMT6*, were fairly significant (GWAS information) for both SHBG_level_-tied sex hormone (total testosterone [rs12406721/r^2^ = 0.86, D′ = 0.93 [42,43]) and lipid metabolism (HDL cholesterol [rs2878349/r^2^ = 0.98, D′ = 1.00] [69], LDL cholesterol [rs111232683/r^2^ = 0.86, D′ = 0.93] [70], BMI [rs12046439/r^2^ = 0.49, D′ = 0.89] [71,72,73]).

In this work, it was found that the allele A rs3779195 (T/A) *BAIAP2L1* increases the risk of UM (by more than 50%) in the BMI ≥ 25 cohort (OR = 1.53). The UM-causal SNP rs3779195 (T/A) *BAIAP2L1* and its highly linked variants define the relationship of the genome position at near *BAIAP2L1* and *BRI3* genes with 86 TFs and regulatory proteins, affect the expression of 15 genes and the splicing of 3 genes including organs implicated in both SHBG production (liver) and UM biology (uterus, ovary, thyroid, adrenal, brain, blood, muscle skeletal, etc.), and have adipose-significant functionality (several SNPs exhibit significant epigenetic effects [located in the area of potential enhancers/promoters, active enhancers/promoters] in various fat cell cultures, affecting the expression of two genes (*RP11-307C18.1*, *BRI3*) and the *BRI3* gene splicing in both visceral and subcutaneous adipose tissue). In GWAS works by Coviello et al. [39] and Harrison et al. [43], the allele A rs3779195 (T/A) *BAIAP2L1* association’s with a lower SHBG_level_ was shown. Thus, SHBG-lowering allele A rs3779195 (GWAS materials) has been linked with a high UM risk (our data [OR = 1.53]). Importantly, several proxy loci of rs3779195 (T/A) *BAIAP2L1* were involved in the pathways (GWAS information) of SHBG_level_ (rs1688606/r^2^ = 0.96, D′ = 1.00; rs112758337/r^2^ = 0.96, D′ = 1.00; rs4268041/r^2^ = 0.99, D′ = 1.00 [43]), SHBG_level_-tied sex hormone (total testosterone [rs1635612/r^2^ = 0.96, D′ = 1.00] [43]; rs35903783/r^2^ = 0.41, D′ = 1.00 [42]), lipid metabolism (total/LDL cholesterol, apoB [rs112758337/r^2^ = 0.96, D′ = 1.00] [70,74], lipid/lipoprotein (total, HDL) diameter/measurement/ratio ([rs6465679/r^2^ = 0.84, D′ = 1.00] [75]), and body fat percentage ([rs35903783/r^2^ = 0.41, D′ = 1.00] [76]).

So, the results obtained by us in silico persuasively testify to, on the one hand, the expressed functionality of UM-causal loci and their proxy SNPs in the body as a whole, the liver (the main site of SHBG formation), adipose tissue, etc., all of which are organs important for UM biology. Meanwhile, the functionality of the UM-associated locus in women BMI ≥ 25 [rs3779195 (T/A) *BAIAP2L1*] was significantly more pronounced (influences the DNA affinity to 86 TFs and regulatory proteins, affects the 15 genes expression and 3 genes splicing) than the UM-correlated locus in women BMI < 25 [rs17496332 (A/G) *PRMT6*] (exerts the DNA affinity to 59 TFs, affects the only one gene [*PRMT6*] expression). On the other hand, they show the pronounced involvement of the genome regions where UM-causal loci are located in the regulation of the SHBG_level_, SHBG_level_-tied sex hormone (total testosterone) and lipid metabolism, which may be a good biomedical basis for the BMI-dependent differences in the associations of SHBG_level_-tied loci with UM in the studied group of women.

Summarizing the data obtained in our work on two SHBG_level_-tied loci (rs17496332 (A/G) *PRMT6* and rs3779195 (T/A) *BAIAP2L1*) associated with UM (in groups of women with BMI < 25 and BMI ≥ 25 appropriately), the following general pattern can be noted: the SHBG-lowering genetic variant (allele A rs3779195 (T/A) *BAIAP2L1*) has risky values for UM and the SHBG-increasing variant (allele G rs17496332 (A/G) *PRMT6*) has a protective effect on UM (Figure 3). It is well known that SHBG is a transporter of testosterone (to a greater extent) and estrogens (to a lesser extent); therefore, by regulating the level of bioavailable (unrelated to SHBG and therefore bioactive [free hormone hypothesis] [77]) testosterone/estrogens in the body, SHBG (its level) can significantly affect the UM pathophysiology. Numerous previously obtained experimental data indicate the UM risk value of high levels of testosterone and estrogens [78,79]. It is believed that estrogens (by influencing their specific receptors, ER) potentiate the growth of UM (“activate” the proliferation of uterus smooth muscle tissue) [78,79]. Similarly, testosterone can act as a “driver” of myomatous cell growth, the conversion of which into estrogens under the action of a special enzyme, aromatase (actively occurs both in UM and in adipose), contributes to this process [78,79]. In Wang et al.’s work (a sample of FibroGENE dataset, including 20,406 UM and 223,918 controls, was analyzed using the MR method), a causal genetic relationship between a higher SHBG_level_ and a lower UM risk was found [80], which is completely consistent with our results. Along with this, it is important to highlight that studies of BMI-dependent correlations of SHBG_level_-tied polymorphisms with UM have not been conducted so far, and our work is the first on this topic.

It is very important to note the following point: progesterone is one of the key metabolic precursors of androgens and estrogens in the organism [81], which, according to literature data, may be involved in UM pathophysiology [82,83,84,85,86]. In the work of Ruth et al., significant positive correlations between progesterone level, content of dehydroepiandrosterone sulfate (DHEAS) (r = 0.60), testosterone (r = 0.44), free androgen index (FAI, calculated as testosterone/SHBG × 100) (r = 0.39) and, to a lesser extent, the concentration of estradiol (r = 0.17) were shown [41]. Interestingly, the GWAS results obtained by Ruth et al. indicate the presence of common genetic determinants of DHEAS (rs148982377) and progesterone (rs34670419) levels (polymorphisms rs148982377 and rs34670419 are strongly linked [r^2^ = 1.00, D′ = 1.00], located 56 kb apart in the region of *CYP3A4/CYP3A7* genes involved in the steroid biosynthesis pathway) [41]. Therefore, the UM-significant SHBG_level_-tied effects of genetic polymorphisms, realized through testosterone and estrogens, described in our work, may to a certain extent be mediated by the effects of their precursor—progesterone. Progesterone, interacting similarly as with its specific receptors (progesterone receptors, PRs), and with non-genomic membrane receptors (mPRs/PGRMCs), activates a number of signaling pathways (WNT/β-catenin, PI3K/AKT pathways) that stimulate the growth/proliferation of myomatous cells, promote their survival (by reducing apoptosis), lead to certain vascular changes that improve blood supply to fibroids and cause UM-significant modification of the extracellular matrix (it is a key component of the tumor structure) [82,83,84,85,86,87,88,89]. It should be noted that estrogens and progesterone act together during UM formation: estrogens in tumor cells cause an increase in the PR expression, which makes UM more “sensitive” to the signals of these hormones [82]. Animal models have shown that the PR expression level in myomatous nodes is higher than that of estrogen receptors [90]. In a study performed by Khan et al., it was found that, in women with UM who did not receive GnRH agonist therapy, the PR content was significantly higher than that of estrogen receptors [91]. It is noteworthy that mitotic activity in myomatous cells is higher during the secretory phase of the menstrual cycle (when progesterone dominates) than during the proliferative phase (when estrogens dominate) [92].

The SHBG_level_ in the body is BMI-dependent: in obese and overweight individuals, SHBG_level_ is significantly reduced [77]. Therefore, BMI can be a significant modifier for SHBG_level_-tied effects in the body, which we have established in our work in relation to UM: in women with a BMI < 25, the susceptibility to UM correlates with the rs17496332 (A/G) *PRMT6*, whereas in women with a BMI ≥ 25, the predisposition to UM depends on the rs3779195 (T/A) *BAIAP2L1*. Along with that, the orientation of SHBG_level_-tied loci associations with UM was the same in both groups differing in BMI: the SHBG-lowering allele was UM risky, and the SHBG-decreasing allele, on the contrary, was UM protective, which may point out the “universal” nature of the connection between SHBG and UM (Figure 3). Interestingly, in a series of previous genetic studies in the same population (Europeans of Central Russia), we found cogent acknowledgement of the crucial role of obesity/overweight as a modifier of genetic variants associations with sex hormone/SHBG-related pathologies such as breast cancer [93,94,95], osteoarthritis [96], and preeclampsia [97].

It is very interesting that this panel of polymorphisms (nine SHBG_level_-tied loci) was used by us earlier in a study of two diseases such as breast cancer [98] and endometriosis [99]. According to the previously obtained data, susceptibility to breast cancer is determined by the rs10454142 *PPP1R21* [98], and predisposition to endometriosis was determined by the rs440837 *ZBTB10* [99]. The results of this study and the data we previously obtained indicate the presence of pronounced specific features of the involvement of SHBG_level_-tied loci in the formation of various hormone-dependent diseases of the female reproductive system, which should be taken into account when planning further genetic studies of these diseases using other SHBG_level_-tied markers, as well as when determining the prospects for using SHBG_level_-tied variants in practice medicine (gynecology, oncology).

One of the limitations of this study is the lack of information about certain characteristics of the studied patient/control groups (such as diet and education level) that may influence the results of genetic analysis.

## 5. Conclusions

As the results obtained for the first time in this work showed, the causal value for UM of the functionally weighty SHBG_level_-tied polymorphisms was BMI-conditioned: UM risk was determined by the rs17496332 (A/G) *PRMT6* in the BMI < 25 group and rs3779195 (T/A) *BAIAP2L1* in the BMI ≥ 25 cohort.

## Figures and Tables

**Table 1:** Phenotypic characteristics of the study participants.

Parameters	BMI ≥ 25			BMI < 25		
	Cases $\bar{x}$ ± SD/% (n)	Controls $\bar{x}$ ± SD/% (n)	*p*	Cases $\bar{x}$ ± SD/% (n)	Controls $\bar{x}$ ± SD/% (n)	*p*
*N*	379	403	-	190	570	-
Age, years	45.05 ± 7.78	44.07 ± 8.27	**<0.05**	39.58 ± 8.27	35.28 ± 8.13	**<0.001**
BMI, kg/m^2^	30.66 ± 4.29	28.51 ± 3.88	**<0.001**	21.99 ± 1.82	21.42 ± 1.83	>0.05
Family history of uterine myoma (mother had uterine leiomyoma)	35.36 (134)	19.11 (77)	**<0.001**	34.74 (66)	15.61 (89)	**<0.001**
Married	85.22 (323)	85.86 (346)	>0.05	84.74 (161)	85.96 (490)	>0.05
Smoker (yes)	13.72 (52)	15.14 (61)	>0.05	13.68 (26)	18.42 (105)	>0.05
Drinking alcohol (≥7 drinks per week)	2.90 (11)	1.74 (7)	>0.05	3.16 (6)	4.04 (23)	>0.05
Oral contraceptive use	9.50 (36)	10.17 (41)	>0.05	9.47 (18)	10.00 (57)	>0.05
Age at first oral contraceptive use (mean, years)	23.51 ± 2.39	23.72 ± 2.37	>0.05	23.32 ± 2.29	23.54 ± 2.32	>0.05
Age at menarche and menstrual cycle						
Age at menarche, years	13.41 ± 1.31	13.09 ± 1.23	>0.05	13.57 ± 1.32	13.36 ± 1.27	>0.05
Duration of bleeding menstrual (mean, days)	5.24 ± 1.68	4.94 ± 0.94	>0.05	5.05 ± 1.45	4.97 ± 0.96	>0.05
Menstrual cycle length (mean, days)	27.94 ± 2.26	28.04 ± 2.26	>0.05	28.27 ± 1.80	28.20 ± 2.24	>0.05
Reproductive characteristic						
Age at first birth (mean, years)	21.06 ± 2.35	21.57 ± 3.44	>0.05	21.58 ± 3.20	21.72 ± 3.42	>0.05
No of gravidity (mean)	3.64 ± 2.20	2.63 ± 1.56	**<0.001**	2.73 ± 2.10	2.23 ± 1.51	**<0.01**
No of births (mean)	1.58 ± 0.80	1.71 ± 0.68	**<0.05**	1.20 ± 0.90	1.41 ± 0.63	**<0.05**
No of spontaneous abortions (mean)	0.29 ± 0.69	0.22 ± 0.48	>0.05	0.18 ± 0.49	0.23 ± 0.49	>0.05
No of induced abortions (mean)	1.73 ± 1.69	0.88 ± 0.90	**<0.001**	1.31 ± 1.50	0.48 ± 0.91	**<0.001**
No of stillbirths	0.01 ± 0.08	0.02 ± 0.14	>0.05	0.01 ± 0.08	0.01 ± 0.11	>0.05
History of infertility	13.72 (52)	5.21 (21)	**<0.001**	13.68 (26)	5.09 (29)	**<0.01**
Gynecological pathologies						
Cervical disorders	27.97 (106)	28.54 (115)	>0.05	22.11 (42)	22.81 (130)	>0.05
History of sexually transmitted disease	26.91 (102)	26.55 (107)	>0.05	27.37 (52)	27.19 (155)	>0.05
Chronic endometritis	11.87 (45)	7.20 (29)	**<0.05**	6.32 (12)	4.56 (26)	>0.05
Chronic inflammation of adnexa	35.88 (136)	34.24 (138)	>0.05	32.11 (61)	30.35 (173)	>0.05
Endometrial hyperplasia	47.23 (179)	-	**-**	46.84 (89)	-	**-**
Endometriosis	35.36 (134)	-	**-**	38.42 (73)	-	**-**
Adenomyosis	20.32 (77)	-	**-**	23.16 (44)	-	**-**

*p* values < 0.05 are shown in bold.

**Table 2:** Data on the nucleotide sequences of primers and probes used for genotyping of the SHBG_level_-tied loci.

Chr	SNP	Gene	Nucleotide Sequences of Primers and Probes
1	rs17496332	*PRMT6*	F: AGCCTTGAAAGAGTGTATA R: GTGAGAATGTTCCTTGTG FAM-acaaAaCaTaGtAtctgc-BHQ-1 VIC-acaaAaCaCaGtAtctgc-BHQ-2
2	rs780093	*GCKR*	F: GCCGTTGCTCTCATTCTTA R: CCTTCTTCCACCACCATC FAM-cctGgtTggGggc-BHQ-1 VIC-cctGgtCggGggc-BHQ-2
2	rs10454142	*PPP1R21*	F: CCTGCTCTGTATATCTTC R: GTTCCTCTATACATTCATATG FAM-cttacTaaTggCctcc-BHQ-1 VIC-cttacTaaCggCctcc-BHQ-2
7	rs3779195	*BAIAP2L1*	F: CGAGAGCACTTTCAACTA R: CCAGGCTTTACTGAGAAA FAM-atttctTgaTttTggggag-BHQ-1 VIC-atttctTgaAttTggggag-BHQ-2
8	rs440837	*ZBTB10*	F: CAAGCAAAAATATTGTGAAA R: GAAGGATAGAGTTAATGGA FAM-aattatCtGtTtAgAatttatt-BHQ-1 VIC-aattatCtGtCtAgAatttatt-BHQ-2
10	rs7910927	*JMJD1C*	F: CACTGACTTCTTAAAAAAG R: TGCAGGTATTTGATATAAC FAM-tgcatAtAaAtTtTctatttta-BHQ-1 VIC-tgcatAtAaCtTtTctatttta-BHQ-2
12	rs4149056	*SLCO1B1*	F: ACACCATATTGTCAAAGTTTG R: GCGAAATCATCAATGTAAGAA FAM-tggataTaTgTgTtCatggg-BHQ-1 VIC-tggataTaTgCgTtCatggg-BHQ-2
15	rs8023580	*NR2F2*	F: CAAGGAAATATACTTCTTATTCATA R: CCAAGTGGAAATTATTGTC FAM-aagaatTcTaTgTtTagattt-BHQ-1 VIC-aagaatTcTaCgTtTagattt-BHQ-2
17	rs12150660	*SHBG*	F: GCTGGTCTCAAACTCCTC R: GAGGTAAATTTGTTGGGAACTTA FAM-agccactTcgCccg-BHQ-1 VIC-agccactGcgCccg-BHQ-2

F, the initial primer; R, the reverse primer.

**Table 3:** Associations of the studied gene polymorphisms with uterine myoma among BMI < 25 and BMI ≥ 25 female.

Chr	SNP	Gene	Minor Allele	n	Allelic Model				Additive Model				Dominant Model				Recessive Model			
					OR	95%CI		*p*	OR	95%CI		*p*	OR	95%CI		*p*	OR	95%CI		*p*
						L95	U95			L95	U95			L95	U95			L95	U95	
female with BMI < 25																				
1	rs17496332	*PRMT6*	G	711	0.82	0.64	1.06	0.127	**0.70**	**0.51**	**0.94**	**0.023**	0.71	0.48	1.05	0.084	0.52	0.27	1.01	0.055
2	rs780093	*GCKR*	T	727	1.16	0.91	1.47	0.232	1.13	0.85	1.50	0.384	1.25	0.82	1.90	0.303	1.08	0.65	1.81	0.764
2	rs10454142	*PPP1R21*	C	717	0.92	0.71	1.19	0.520	0.94	0.71	1.26	0.699	0.90	0.61	1.33	0.602	1.00	0.54	1.85	0.999
7	rs3779195	*BAIAP2L1*	A	717	0.91	0.66	1.26	0.564	1.03	0.71	1.05	0.878	1.11	0.73	1.69	0.630	0.46	0.09	2.34	0.350
8	rs440837	*ZBTB10*	G	706	1.15	0.87	1.51	0.333	1.30	0.94	1.78	0.111	1.28	0.86	1.90	0.225	1.82	0.85	3.09	0.123
10	rs7910927	*JMJD1C*	T	726	0.89	0.70	1.13	0.329	0.87	0.66	1.14	0.303	0.89	0.59	1.36	0.591	0.75	0.46	1.21	0.239
12	rs4149056	*SLCO1B1*	C	690	0.93	0.69	1.24	0.602	0.89	0.63	1.24	0.486	0.88	0.59	1.32	0.542	0.78	0.31	1.97	0.604
15	rs8023580	*NR2F2*	C	720	0.94	0.72	1.23	0.663	1.03	0.76	1.39	0.846	1.13	0.76	1.67	0.542	0.80	0.39	1.62	0.536
17	rs12150660	*SHBG*	T	731	0.97	0.74	1.29	0.846	0.98	0.71	1.33	0.875	0.99	0.67	1.46	0.952	0.90	0.41	1.98	0.784
female with BMI ≥ 25																				
1	rs17496332	*PRMT6*	G	741	1.01	0.82	1.25	0.902	1.06	0.86	1.34	0.548	1.03	0.75	1.41	0.859	1.24	0.80	1.92	0.345
2	rs780093	*GCKR*	T	743	1.06	0.86	1.31	0.559	1.06	0.85	1.33	0.586	1.04	0.75	1.44	0.801	1.16	0.76	1.77	0.483
2	rs10454142	*PPP1R21*	C	728	1.09	0.87	1.36	0.461	1.07	0.83	1.37	0.604	1.04	0.76	1.43	0.802	1.24	0.70	2.20	0.457
7	rs3779195	*BAIAP2L1*	A	735	1.19	0.92	1.55	0.184	1.27	0.95	1.68	0.104	**1.53**	**1.06**	**2.09**	**0.018**	0.58	0.24	1.42	0.232
8	rs440837	*ZBTB10*	G	717	1.04	0.81	1.33	0.756	1.04	0.80	1.34	0.785	0.88	0.64	1.22	0.448	2.15	1.09	4.25	0.027
10	rs7910927	*JMJD1C*	T	745	1.14	0.93	1.39	0.216	1.12	0.89	1.40	0.321	1.00	0.69	1.44	0.996	1.35	0.94	1.95	0.106
12	rs4149056	*SLCO1B1*	C	728	1.04	0.81	1.32	0.781	1.05	0.80	1.37	0.753	1.07	0.78	1.48	0.669	0.95	0.45	2.02	0.900
15	rs8023580	*NR2F2*	C	731	1.07	0.85	1.34	0.576	1.00	0.78	1.28	0.983	1.04	0.76	1.42	0.823	0.90	0.50	1.61	0.717
17	rs12150660	*SHBG*	T	755	0.97	0.77	1.22	0.775	0.92	0.72	1.19	0.533	0.95	0.69	1.29	0.721	0.76	0.41	1.44	0.404

All results were obtained after adjustment for covariates; OR, odds ratio; 95% CI, 95% confidence interval; *p*_perm_ values < 0.025 are shown in bold.

**Table 4:** Probable functionality of the UM-correlated locus rs17496332 (A/G) *PRMT6* and proxy SNPs (r ≥ 0.80) in liver and adipose tissue (in silico data).

SNP (Position hg38) (r^2^, LD)	Haploreg Data		GTE-Portal Data		
	Transcription Factors	Adipose-Derived Mesenchymal Stem Cell Cultured Cells	Liver	Visceral Adipose	Subcutaneous Adipose
rs113329442 (106996630) (r^2^ = 0.99, LD = 1.00)	Brachyury, GR, Irf, PU.1, Sox		*PRMT6*	*PRMT6*	*PRMT6*
rs3861909 (107001554) (r^2^ = 0.97, LD = −0.99)	AP-1, Pdx1, RORalpha1	H3K4me1_Enh	*PRMT6*	*PRMT6*	*PRMT6*
rs17496332 (107003753)	DMRT1, FAC1		*PRMT6*	*PRMT6*	*PRMT6*
rs2878349 (107006623) (r^2^ = 0.98, LD = 1.00)			*PRMT6*	*PRMT6*	*PRMT6*
rs5776878 (107008396) (r^2^ = 0.98, LD = −1.00)	AP-1, Cart1, HDAC2, Zfp105		*	*	*
rs72697623 (107011647) (r^2^ = 0.98, LD = 1.00)	CEBPA, CEBPB, p300	H3K4me1_Enh	*PRMT6*	*PRMT6*	*PRMT6*
rs4914939 (107015739) (r^2^ = 0.94, LD = 0.99)	Cdc5, Fox, Foxa, Foxf1, Foxi1, Foxj1, Foxj2, Foxl1, Foxp1, HDAC2, Mef2, Pou2f2, TATA, Zfp105, p300	H3K4me1_Enh	*	*	*
rs12406721 (107020621) (r^2^ = 0.91, LD = 0.96)	EWSR1-FLI1, HDAC2, Hoxa5		*PRMT6*	*PRMT6*	*PRMT6*
rs61798463 (107023312) (r^2^ = 0.88, LD = 0.96)	IRC900814		*PRMT6*	*PRMT6*	*PRMT6*
rs111232683 (107023527) (r^2^ = 0.85, LD = 0.93)	CACD, CCNT2, CHD2, Ets, Egr-1, GR, Klf4, Myc, NRSF, PU.1, Pax-4, Pou2f2, RREB-1, SP1, SREBP, Spz1, STAT, ZNF219, Zfp281, Zfp740, UF1H3BETA		*	*	*
rs56111229 (107024067) (r^2^ = 0.85, LD = 0.93)	AP-1, Arid3a, Bach1, Bsx, GATA, GR, KAP1, Zfp691		*PRMT6*	*PRMT6*	*PRMT6*
rs55924375 (107024068) (r^2^ = 0.85, LD = 0.93)	AP-1, Arid3a, Bach1, Bsx, GATA, GR, HNF1, Hoxb4, KAP1, Zfp691		*PRMT6*	*PRMT6*	*PRMT6*
rs61798468 (107026694) (r^2^ = 0.88, LD = 0.96)	Arid3a, Pou2f2, Sox, Zfp105		*PRMT6*	*PRMT6*	*PRMT6*
rs200443569 (107028138) (r^2^ = 0.81, LD = 0.91)	GATA, HDAC2, Ik-2, NF-AT, Sox, TATA		*	*	*
rs72442711 (107028139) (r^2^ = 0.81, LD = 0.90)	Foxp1, GATA, HDAC2, Irf, Sox, TATA		*	*	*

* The information in the GTE-portal database is not provided; H3K4me1_Enh, SNP location in the region of H3K4me1 histones marking enhancers; UM-correlated locus is highlighted in gray.

**Table 5:** Probable functionality of the UM-correlated locus rs3779195 (T/A) *BAIAP2L1* and proxy SNPs (r ≥ 0.80) in liver, adipose and uterus (in silico data).

SNP (Position hg38) (r^2^, LD)	Haploreg Data					GTE-Portal Data (eQTL/sQTL)			
	Transcription Factors/Proteins Bound	Liver	Adipocyte Cultured Cells						
			Mesenchymal Stem Cell-Derived Adipocyte Cultured Cells	Adipose-Derived Mesenchymal Stem Cell Cultured Cells	Adipose Nuclei	Visceral Adipose	Subcutaneous Adipose	Liver	Uterus
rs6950023 (98286323) (r^2^ = 0.90, LD = −0.96)	Nkx3/POL24H8, AP2ALPHA, AP2GAMMA, CMYC, GTF2F1, MAX, MXI1, POL2, PRDM1, PU1	H3K4me1_Enh H3K4me3_Pro H3K27ac_Enh H3K9ac_Pro	H3K4me1_Enh H3K4me3_Pro H3K9ac_Pro	H3K4me1_Enh H3K4me3_Pro H3K9ac_Pro	H3K4me1_Enh H3K4me3_Pro H3K27ac_Enh H3K9ac_Pro	*RP11-307C18.1*, *BRI3/BRI3*	*RP11-307C18.1*, *BRI3/BRI3*	*RP11-307C18.1*, *BRI3*	*RP11-307C18.1*
rs6967728 (98286325) (r^2^ = 0.90, LD = −0.96)	Nkx3/POL24H8, AP2ALPHA, AP2GAMMA, CEBPB, CMYC, GTF2F1, MAX, MXI1, POL2, PRDM1, PU1	H3K4me1_Enh H3K4me3_Pro H3K27ac_Enh H3K9ac_Pro	H3K4me1_Enh H3K4me3_Pro H3K9ac_Pro	H3K4me1_Enh H3K4me3_Pro H3K9ac_Pro	H3K4me1_Enh H3K4me3_Pro H3K27ac_Enh H3K9ac_Pro	*RP11-307C18.1*, *BRI3/BRI3*	*RP11-307C18.1*, *BRI3/BRI3*	*RP11-307C18.1*, *BRI3*	*RP11-307C18.1*
rs7015 (98291311) (r^2^ = 0.85, LD = −0.97)	Dbx1, Hoxa10, Hoxa9, Hoxb13, Hoxd10, Ncx, Pou3f2, Sox, Zfp105	H3K4me1_Enh		H3K9ac_Pro	H3K4me1_Enh H3K27ac_Enh H3K9ac_Pro	*RP11-307C18.1*, *BRI3/BRI3*	*RP11-307C18.1*, *BRI3/BRI3*	*RP11-307C18.1*, *BRI3*	*RP11-307C18.1*
rs13232861 (98299769) (r^2^ = 0.81, LD = −0.96)	AP-1, AP-2, BAF155, BATF, Bach1, Bach2, CHD2, E2F, Egr-1, GATA, GR, HMGN3, KAP1, NRSF, Nrf1, PRDM1, SRF, STAT, Sin3Ak-20, TCF4, Zfp161, p300	H3K27ac_Enh				*RP11-307C18.1*, *BRI3/BRI3*	*RP11-307C18.1*, *BRI3/BRI3*	*RP11-307C18.1*, *BRI3*	*RP11-307C18.1*
rs11290747 (98300261) (r^2^ = 0.95, LD = −0.97)	CHD2, CTCFL, E2F, GR, NF-kappaB, NRSF, Rad21, SP1, UF1H3BETA, ZNF263, Znf143, p300	H3K4me1_Enh H3K27ac_Enh H3K9ac_Pro				*RP11-307C18.1*, *BRI3/BRI3*	*RP11-307C18.1*, *BRI3/BRI3*	*RP11-307C18.1*, *BRI3*	*RP11-307C18.1*
rs2906184 (98310675) (r^2^ = 0.95, LD = 0.97)	Arid3a, CEBPG, Dbx1, HDAC2, Ncx, PLZF, TATA, Zfp105					*	*	*	*
rs1635609 (98320502) (r^2^ = 0.96, LD = −0.98)	HNF1, Hoxa4, Pax-4, Pax-6, Pou2f2					*RP11-307C18.1*, *BRI3/BRI3*	*RP11-307C18.1*, *BRI3/BRI3*	*RP11-307C18.1*, *BRI3*	*RP11-307C18.1*
rs1688607 (98322009) (r^2^ = 0.92, LD = −0.98)	GR, VDR	H3K4me1_Enh			H3K4me1_Enh	*RP11-307C18.1*, *BRI3/BRI3*	*RP11-307C18.1*, *BRI3/BRI3*	*RP11-307C18.1*, *BRI3*	*RP11-307C18.1*
rs1688606 (98345539) (r^2^ = 0.98, LD = −0.99)	GATA					*RP11-307C18.1*, *BRI3/BRI3*	*RP11-307C18.1*, *BRI3/BRI3*	*RP11-307C18.1*, *BRI3*	*RP11-307C18.1*
rs112758337 (98347956) (r^2^ = 0.98, LD = 0.99)	MAZ, MAZR, MZF1:1–4					*RP11-307C18.1*, *BRI3/BRI3*	*RP11-307C18.1*, *BRI3/BRI3*	*RP11-307C18.1*, *BRI3*	*RP11-307C18.1*
rs77032872 (98355009) (r^2^ = 0.98, LD = 0.99)	Foxl1, HNF1, Mef2, Nkx2, Pax-2, TATA	H3K4me1_Enh H3K27ac_Enh	H3K4me1_Enh H3K9ac_Pro	H3K4me1_Enh	H3K4me1_Enh H3K27ac_Enh	*RP11-307C18.1*, *BRI3/BRI3*	*RP11-307C18.1*, *BRI3/BRI3*	*RP11-307C18.1*, *BRI3*	*RP11-307C18.1*
rs12704986 (98357118) (r^2^ = 0.97, LD = −0.99)	EBF, HNF4, Nr2f2, RXRA	H3K27ac_Enh				*	*	*	*
rs3779196 (98360794) (r^2^ = 0.98, LD = −0.99)	Ascl2, BHLHE40, CEBPB, CTCF, Lmo2-complex, TCF12/USF1, CTCF, RAD21, SMC3, GABP, HDAC2, MAFK, POL2	H3K4me1_Enh H3K27ac_Enh H3K9ac_Pro				*RP11-307C18.1*, *BRI3/BRI3*	*RP11-307C18.1*, *BRI3/BRI3*	*RP11-307C18.1*, *BRI3*	*RP11-307C18.1*
rs6965424 (98361813) (r^2^ = 0.98, LD = −0.99)		H3K4me1_Enh				*RP11-307C18.1*, *BRI3/BRI3*	*RP11-307C18.1*, *BRI3/BRI3*	*RP11-307C18.1*, *BRI3*	*RP11-307C18.1*
rs3779195 (98364050)	Foxp1					*RP11-307C18.1*, *BRI3/BRI3*	*RP11-307C18.1*, *BRI3/BRI3*	*RP11-307C18.1*, *BRI3*	*RP11-307C18.1*
rs4268041 (98376226) (r^2^ = 0.91, LD = −0.98)	Rad21					*RP11-307C18.1*, *BRI3/BRI3*	*RP11-307C18.1*, *BRI3/BRI3*	*RP11-307C18.1*, *BRI3*	*RP11-307C18.1*
rs201244010 (98378225) (r^2^ = 0.95, LD = −0.99)						*	*	*	*
rs5886063 (98378229) (r^2^ = 0.95, LD = −0.98)	BATF, FAC1, MAZ, Myc, Pax-2					*RP11-307C18.1*, *BRI3/BRI3*	*RP11-307C18.1*, *BRI3/BRI3*	*RP11-307C18.1*, *BRI3*	*RP11-307C18.1*
rs10953259 (98383795) (r^2^ = 0.95, LD = −0.98)	DMRT4, Lhx3, Pou6f1	H3K4me1_Enh H3K9ac_Pro				*RP11-307C18.1*, *BRI3/BRI3*	*RP11-307C18.1*, *BRI3/BRI3*	*RP11-307C18.1*, *BRI3*	*RP11-307C18.1*
rs13310668 (98393841) (r^2^ = 0.88, LD = −0.94)						*RP11-307C18.1*, *BRI3/BRI3*	*RP11-307C18.1*, *BRI3/BRI3*	*RP11-307C18.1*, *BRI3*	*RP11-307C18.1*
rs10953260 (98404180) (r^2^ = 0.93, LD = −0.96)	PU1					*RP11-307C18.1*, *BRI3/BRI3*	*RP11-307C18.1*, *BRI3/BRI3*	*RP11-307C18.1*, *BRI3*	*RP11-307C18.1*

* The information in the GTE-portal database is not provided; H3K4me1_Enh, SNP location in the region of H3K4me1 histones marking enhancers; H3K27ac_Enh, active enhancers; H3K4me3_Pro, promoters; H3K9ac_Pro, active promoters; UM-correlated locus is highlighted in gray.

## Data Availability

The authors declare that the data supporting the findings of this study are available within the paper and its Supplementary Tables. The raw data used in this study can be obtained from the corresponding author upon reasonable request.

## Supplementary Materials

The following supporting information can be downloaded at: https://www.mdpi.com/article/10.3390/life15091459/s1, Table S1: The GWAS data about associations of the studied candidate gene polymorphisms with the circulating SHBG and other sex hormone concentrations; Table S2: The regulatory potential of the studied SNPs; Table S3: The allele and genotype frequencies of the studied SNPs in the uterine myoma and control groups with BMI < 25; Table S4: The allele and genotype frequencies of the studied SNPs in the uterine myoma and control groups with BMI ≥ 25; Table S5: Regulatory effects of the UM-associated loci and SNPs in high LD (r^2^ ≥ 0.80); Table S6: The eQTL effects of the UM-associated SNPs in various tissues/organs; Table S7: eQTL values of SNPs in high LD (r^2^ ≥ 0.80) with the UM-associated polymorphisms; Table S8: The sQTL effects of the UM-associated SNP in various tissues/organs; Table S9: sQTL values of SNPs in high LD (r^2^ ≥ 0.80) with the UM-associated polymorphism; Table S10: Gene set enrichment analysis of biological pathways correlated with 59 TFs and PRMT6 protein functionally related to the UM-associated rs17496332 (A/G) PRMT6 and their proxy 14 SNPs; Table S11: Gene set enrichment analysis of biological pathways correlated with 86 protein-regulatory/TFs and 16 protein functionally related to UM-associated rs3779195 (T/A) *BAIAP2L1* and their proxy 20 SNPs; Table S12: Molecular/biochemical functions of the TFs and PRMT6 protein functionally related to the UM-associated rs17496332 (A/G) PRMT6 and their proxy 14 SNPs (according to the STRING data); Table S13: Molecular/biochemical functions of the TFs and 15 proteins functionally related to the UM-associated rs3779195 (T/A) BAIAP2L1 and their proxy 20 SNPs (according to the STRING data). Reference [100] is mentioned in Supplementary Materials.

## Abbreviations

The following abbreviations are used in this manuscript:

UM	Uterine fibroids
SHBG	Sex hormone-binding globulin
SHBG_level_	Sex hormone-binding globulin level
SNP	Single-nucleotide polymorphism
GWAS	Genome-wide association studies
BMI	Body mass index
DNA	Deoxyribonucleic acid
LD	Linkage disequilibrium
TFs	Transcription factors
