# Supplementary material for: Obesity/Overweight as a Meaningful Modifier of Associations Between Gene Polymorphisms Affecting the Sex Hormone-Binding Globulin Content and Uterine Myoma
Source: Life (Basel). 2025 Sep 17;15(9):1459. doi: 10.3390/life15091459 (PMC12471284; doi:10.3390/life15091459)
Supplement: Supplementary file 1 [file life-15-01459-s001.zip › life-3817839-supplementary/Suppl Tables/++Suppl table 1.pdf]

Supplementary Table S1. The GWAS data about associations of the studied candidate gene polymorphisms with the circulating SHBG and other sex hormone concentrations.

SNP, gene	Chromosome position (hg38)	Phenotype	Association (significance) (affected allele)	Reference
rs17496332 *PRMT6*	1p13.3 (107003753)	SHBG	β=-0.028 (p=1×10^{-11}) (A)	[39]
rs780093 *GCKR*	2p23.3 (27519736)	SHBG	β=-0.032 (p=2×10^{-16}) (T)	[39]
rs10454142 *FOXN2*	2p16.3 (48419260)	SHBG	β=0.026 (p=1×10^{-7}) (T)	[39]
rs3779195 *BAIAP2L1*	7q21.3 (98364050)	SHBG	β=-0.033 (p=3×10^{-8}) (A)	[39]
		SHBG (women, pre-menopause)	β=-2.41 (p=9×10^{-09}) (A)	[43]
rs440837 *ZBTB10*	8q21.13 (80549739)	SHBG	β=-0.030 (p=3×10^{-9}) (A)	[39]
		SHBG (women, post-menopause)	β=1.43 (p=1×10^{-12}) (G)	[43]
		SHBG (men)	β=0.57 (p=8×10^{-9}) (G)	[43]
rs7910927 *JMJD1C*	10q21.3 (63379150)	SHBG	β=-0.048 (p=6×10^{-35}) (T)	[39]
rs4149056 *SLCO1B1*	12p12.1 (21178615)	SHBG	β=0.029 (p=2×10^{-8}) (T)	[39]
		low testosterone levels (men)	OR=1.14 (p=7×10^{-16}) (C)	[100]
		testosterone (women)	β=0.028 (p=5×10^{-10}) (C)	[44]
		SHBG (women)	β=-0.065 (p =5×10^{-48}) (C)	[44]
		SHBG (women, pre-menopause)	β=-0.062 (p=8×10^{-11}) (C)	[44]
		SHBG (women, post-menopause)	β=-0.079 (p=7×10^{-34}) (C)	[44]
		bioavailable testosterone (women)	β=0.02 (p=2×10^{-16}) (C)	[43]
		SHBG (men)	β=-1.23 (p=7×10^{-29}) (C)	[43]
		SHBG (women)	β=0.030 (p=1×10^{-73}) (T)	[42]
		SHBG (men)	β=0.032 (p=6×10^{-99}) (T)	[42]
		total testosterone (women)	β=-0.029 (p=1×10^{-14}) (T)	[42]
		bioavailable	β=-0.043 (p=3×10^{-35}) (T)	[42]

		testosterone (women)		
		total testosterone (men)	β=0.054 ($p=1\times10^{-39}$) (T)	[42]
rs8023580 *PPP1R21*	15q26.2 (96165062)	SHBG	β=-0.03 ($p=8\times10^{-12}$) (T)	[39]
		low testosterone levels (men)	OR=1.13 ($p=1\times10^{-19}$) (T)	[100]
rs12150660 *SHBG*	17p13.1 (7618597)	SHBG	β=0.103 ($p=2\times10^{-106}$) (T)	[39]
		SHBG (women)	β=6.14 ($p=1\times10^{-300}$) (T)	[43]
		SHBG (men)	β=3.9 ($p=2\times10^{-75}$) (T)	[38]
		total testosterone (men)	β=31.8 ($p=1\times10^{-41}$) (T)	[38]
