# Supplementary material for: Obesity/Overweight as a Meaningful Modifier of Associations Between Gene Polymorphisms Affecting the Sex Hormone-Binding Globulin Content and Uterine Myoma
Source: Life (Basel). 2025 Sep 17;15(9):1459. doi: 10.3390/life15091459 (PMC12471284; doi:10.3390/life15091459)
Supplement: Supplementary file 1 [file life-15-01459-s001.zip › life-3817839-supplementary/Suppl Tables/++Suppl table 3.pdf]

Supplementary Table S3. The allele and genotype frequencies of the studied SNPs in the uterine myoma and control groups with BMI<25.

Chr	SNP	Gene	Minor allele	Major allele	Minor allele frequency	Number of the studied chromosomes	Genotype distribution*	H_o	H_e	P_{HWE}
Uterine myoma (n=190)										
1	rs17496332	*PRMT6*	G	A	0.327	364	16/87/79	0.478	0.440	0.312
2	rs780093	*GCKR*	T	C	0.439	360	30/98/52	0.544	0.493	0.176
2	rs10454142	*PPP1R21*	C	T	0.325	348	18/77/79	0.443	0.439	1.000
7	rs3779195	*BAIAP2L1*	A	T	0.163	356	2/54/122	0.303	0.273	0.175
8	rs440837	*ZBTB10*	G	A	0.269	342	13/66/92	0.386	0.393	0.846
10	rs7910927	*JMJD1C*	T	G	0.450	362	35/93/53	0.514	0.495	0.655
12	rs4149056	*SLCO1B1*	C	T	0.218	354	9/59/109	0.333	0.340	0.825
15	rs8023580	*NR2F2*	C	T	0.285	348	14/71/89	0.408	0.407	1.000
17	rs12150660	*SHBG*	T	G	0.235	366	11/64/108	0.350	0.360	0.684
Control (n=570)										
1	rs17496332	*PRMT6*	G	A	0.372	1058	72/249/208	0.471	0.467	0.926
2	rs780093	*GCKR*	T	C	0.403	1094	86/269/192	0.492	0.481	0.657
2	rs10454142	*PPP1R21*	C	T	0.344	1086	67/239/237	0.440	0.451	0.569
7	rs3779195	*BAIAP2L1*	A	T	0.176	1078	19/152/368	0.282	0.290	0.462
8	rs440837	*ZBTB10*	G	A	0.243	1070	27/206/302	0.385	0.368	0.347
10	rs7910927	*JMJD1C*	T	G	0.480	1092	132/259/154	0.475	0.499	0.265
12	rs4149056	*SLCO1B1*	C	T	0.231	1026	30/177/306	0.345	0.355	0.534
15	rs8023580	*NR2F2*	C	T	0.297	1092	57/210/279	0.385	0.417	0.066
17	rs12150660	*SHBG*	T	G	0.240	1096	38/187/323	0.341	0.365	0.129

* minor allele homozygotes / heterozygotes / major allele homozygotes
