# Supplementary material for: Obesity/Overweight as a Meaningful Modifier of Associations Between Gene Polymorphisms Affecting the Sex Hormone-Binding Globulin Content and Uterine Myoma
Source: Life (Basel). 2025 Sep 17;15(9):1459. doi: 10.3390/life15091459 (PMC12471284; doi:10.3390/life15091459)
Supplement: Supplementary file 1 [file life-15-01459-s001.zip › life-3817839-supplementary/Suppl Tables/++Suppl table 4.pdf]

**Supplementary Table S4.** The allele and genotype frequencies of the studied SNPs in the uterine myoma and control groups with BMI≥25.

| Chr | SNP | Gene | Minor allele | Major allele | Minor allele frequency | Number of the studied chromosomes | Genotype distribution* | $H_o$ | $H_e$ | $P_{HWE}$ |
|---|---|---|---|---|---|---|---|---|---|---|
| Uterine myoma (n=379) | | | | | | | | | | |
| 1 | rs17496332 | *PRMT6* | G | A | 0.365 | 720 | 53/157/150 | 0.436 | 0.464 | 0.257 |
| 2 | rs780093 | *GCKR* | T | C | 0.408 | 718 | 62/169/128 | 0.471 | 0.483 | 0.662 |
| 2 | rs10454142 | *PPP1R21* | C | T | 0.303 | 696 | 31/149/168 | 0.428 | 0.423 | 0.899 |
| 7 | rs3779195 | *BAIAP2L1* | A | T | 0.202 | 714 | 9/126/222 | 0.353 | 0.322 | 0.098 |
| 8 | rs440837 | *ZBTB10* | G | A | 0.237 | 670 | 27/105/203 | 0.313 | 0.362 | 0.016 |
| 10 | rs7910927 | *JMJD1C* | T | G | 0.514 | 718 | 95/179/85 | 0.499 | 0.500 | 1.000 |
| 12 | rs4149056 | *SLCO1B1* | C | T | 0.234 | 714 | 16/135/206 | 0.378 | 0.358 | 0.375 |
| 15 | rs8023580 | *NR2F2* | C | T | 0.283 | 692 | 25/146/175 | 0.422 | 0.406 | 0.510 |
| 17 | rs12150660 | *SHBG* | T | G | 0.259 | 730 | 21/147/197 | 0.403 | 0.384 | 0.413 |
| Control (n=403) | | | | | | | | | | |
| 1 | rs17496332 | *PRMT6* | G | A | 0.362 | 762 | 56/164/161 | 0.430 | 0.462 | 0.184 |
| 2 | rs780093 | *GCKR* | T | C | 0.393 | 768 | 61/180/143 | 0.469 | 0.477 | 0.749 |
| 2 | rs10454142 | *PPP1R21* | C | T | 0.286 | 760 | 30/157/193 | 0.413 | 0.408 | 0.900 |
| 7 | rs3779195 | *BAIAP2L1* | A | T | 0.175 | 756 | 18/96/264 | 0.254 | 0.288 | 0.030 |
| 8 | rs440837 | *ZBTB10* | G | A | 0.230 | 764 | 15/146/221 | 0.382 | 0.355 | 0.150 |
| 10 | rs7910927 | *JMJD1C* | T | G | 0.482 | 772 | 81/210/95 | 0.544 | 0.499 | 0.084 |
| 12 | rs4149056 | *SLCO1B1* | C | T | 0.228 | 742 | 18/133/220 | 0.359 | 0.352 | 0.770 |
| 15 | rs8023580 | *NR2F2* | C | T | 0.270 | 770 | 32/144/209 | 0.374 | 0.394 | 0.303 |
| 17 | rs12150660 | *SHBG* | T | G | 0.265 | 780 | 29/149/212 | 0.382 | 0.390 | 0.697 |

* minor allele homozygotes / heterozygotes / major allele homozygotes
